# *Ziziphora clinopodioides* Flavonoids Protect Myocardial Cell Damage from Myocardial Ischemia-Reperfusion Injury

**DOI:** 10.1155/2018/8495010

**Published:** 2018-12-03

**Authors:** Qin Li, Dilnur Tursun, Chenyang Shi, Merhaba Heyrulla, Xinyue Zhang, Weijun Yang

**Affiliations:** ^1^Department of Pharmacology, Institute of Materia Medica, Zhejiang Academy of Medical Sciences, Hangzhou, China; ^2^Key Laboratory of Xinjiang Uighur Medicine, Xinjiang Institute of Materia Medical, Urumqi, China

## Abstract

To investigate effects of* Ziziphora clinopodioides *Lam. flavonoids on ischemia-reperfusion injury of myocardial cells. After application of 6.25, 25, and 100 *μ*g/mL* Ziziphora clinopodioides *Lam. flavonoids to H9C2 myocardial cells for 24H, they were treated for 4 hours with hydrogen peroxide to induce oxidative damage, whereas controls were cells without treatment and cells only incubated with hydrogen peroxide. Cell viability, lactate dehydrogenase release and mitochondrial membrane potential, intracellular Na+/K+-ATPase activity and ATP content, and reactive oxygen species formation were monitored. An ischemia-reperfusion injury rat model was established by left anterior descending coronary artery ligature in 48 Sprague-Dawley rats, which were divided into positive control with isosorbide mononitrate (10 mg/kg) injection (n=8), model (ischemia-reperfusion, n=8), sham-operated (n=8), and* Ziziphora clinopodioides *Lam. flavonoids low (75 mg/kg, n=8), medium (150 mg/kg, n=8), and high concentration (300 mg/kg, n=8) groups. Superoxide dismutase activity and malondialdehyde content in homogenized hearts were measured and ischemic and infarction areas were triphenyl tetrazolium chloride and H&E stained for pathological and morphological examinations.* Ziziphora clinopodioides *Lam. flavonoids preconditioning improved cell viability (*P*<0.01), intracellular Na/K ATPase activity (*P*<0.001), and intracellular ATP content (*P*<0.001) and maintained mitochondrial membrane potential (*P*<0.05) in hydrogen peroxide treated H9C2 cells as well as rescued superoxide dismutase activity (*P*<0.01), decreased the malondialdehyde content (*P*<0.001), and reduced myocardial damage in the ischemia-reperfusion rat model (*P*<0.001) compared to the controls.* Ziziphora clinopodioides *Lam. flavonoids may be an effective drug for protecting myocardial tissue from ischemia-reperfusion injury by reducing reactive oxygen species related damage.

## 1. Introduction

The herbal plant* Ziziphora clinopodioides* Lam. (Lamiaceae) (ZC) grows in Mediterranean to central Asian locations, Afghanistan, Russia, Mongolian, and other regions. The subspecies* Ziziphora bungeana* Juz. in China is located only in the gravel slopes, semidesert grasslands, and sandy beaches in Xinjiang, with an altitude of 700-1,100 m. ZC is a perennial shrub plant, used as a traditional Uygur medicine, and widely used to treat hypertension, coronary heart disease, in addition to other cardiovascular and cerebrovascular diseases by the people of Xinjiang [1] and it is also used for the extraction of essential oil. The research of foreign scholars has mainly focused on the composition and pharmacological action of the volatile oil of ZC [2].

Extracts from ZC have been shown to contain a large number of flavonoids and polyphenolic compounds that exhibited protective effects on rat acute myocardial ischemia and neonatal rat cardiomyocytes [3, 4]. Twenty-nine chemical components have been identified in the essential oils obtained from the aerial parts of ZC, when harvested at different growth stages. Pulegone (30.1%), thymol (21.3%),* p*-mentha-3-en-8-ol (12.9%), and piperitenone (9.3%) were the main components detected in the preflowering stage. In the flowering stage, the main constituents were pulegone (44.6%), p-mentha-3-en-8-ol (10.5%), 1,8-cineoil (0.4%), piperitenone (8.7%), and thymol (6.7%). In the postflowering stage, pulegone (41.3%), isomenthone (11.6%),* p*-mentha-3-en-8-ol (11%), p-mentha-3,8-diene (7.2%), and thymol (5.8%) were identified as the major components [5]. Many compounds extracted from ZC have shown biological activity including acacetin, apigenin, chrysin, thymonin, acetovanillone, 4-hydroxyacetophenone, and ethyl 4-coumarate, which induce vasodilator actions* in vitro* on rat isolated thoracic aortic rings [6]. The dichloromethane extracts of ZC (ZCDE) exhibited endothelium-independent vasodilating properties* in vivo* that are mediated by inhibition of extracellular Ca^2+^ influx through voltage- and receptor-operated Ca^2+^ channels (VDDCs and ROCCs), thus inhibiting Ca^2+^ release from intracellular stores; they also stimulate the opening of voltage-dependent K^+^ channels [6].

Damage caused by free radical-induced oxidation in the human body is thought to play a significant role in aging and also contribute to the development of disease. Therefore, an investigation of the active herbal ingredients producing antioxidation has become a worldwide research topic [7]. Herbal antioxidants have been shown to inhibit lipid peroxidation and increase the antioxidation capacity of myocardial cells [8]. During myocardial ischemia-reperfusion, a large quantity of oxygen free radicals are produced in myocardial cells, which trigger lipid peroxidation and the formation of malondialdehyde (MDA), leading to cell membrane damage and an increase in the internal fluxes of Ca^2+^, which results in overloading of intracellular Ca^2+^ stores. Thus, cellular structure and function, such as contraction, enzymatic activity, and metabolism are impaired, which may lead to cell death [9]. MDA is a metabolic product of lipid peroxidation and its intercellular level indirectly reflects the extent of cell damage caused by free radicals. Superoxide dismutase (SOD) is a negatively charged metal protease, which removes the ultra-oxygen anion by disproportionation [10], protecting the body from damage by oxygen free radicals. The level of its enzymatic activity indirectly reflects the ability to remove oxygen free radicals.

It was established that ZCF had an obvious protective effect on pituitrin-induced myocardial ischemia in rats, improved the antioxidant capacity of myocardial cells, and had a protective effect on anoxia reoxygenation of cardiac myocytes. It also had a protective effect on ischemia-reperfusion injury in isolated rat hearts [11]. In the present study, we investigated the protective effect of ZCF on myocardial cells during ischemia-reperfusion.

## 2. Materials and Methods

### 2.1. Ziziphora clinopodioides Flavonoids

Yang Weijun of the Xinjiang Institute of Materia Medical kindly provided the flavonoids used in the present experiments, which were obtained as described in a previous study [4].

### 2.2. Effects of ZCF on a H9C2 Myocardial Cell Injury Model

#### 2.2.1. Culture Conditions of H9C2 Rat Myocardial Cells

H9C2 rat myocardial cells were purchased from the Shanghai Cell Bank of the Chinese Academy of Sciences. Cells were cultured in DMEM containing 10% FBS (Hangzhou GiNo Biological Pharmaceutical Technology Co., Ltd., Hangzhou, China) in a CO_2_ incubator at 37°C and passaged every 3 to 4 days.

#### 2.2.2. Establishment of the H9C2 Myocardial Cell Injury Model

Cells in the logarithmic growth phase were collected and the cell concentration was adjusted to 1 × 10^5^/mL in DMEM containing 10% FBS. Cell suspensions (1 mL aliquots) were inoculated in 24 well plates and the cells allowed growing and adhering to the wall for 24 h.

The cells were divided into groups: normal control group (n=3); model control group (n=3), and ZCF treatment groups with low (6.25 *μ*g/mL) (n=3), medium (25 *μ*g/mL) (n=3), and high (100 *μ*g/mL) (n=3) concentrations of ZCF. After 24 hours, the drug-containing medium was removed from the ZCF-treated cells and replaced by medium containing H_2_O_2_ at a concentration of 0.8 mmol/L, and cells were then incubated for 4 h to establish the H9C2 myocardial ischemia-reperfusion model. Cells in the normal control group were not incubated with H_2_O_2_ or ZCF and the model control group was only challenged with H_2_O_2_ also without ZCF application.

#### 2.2.3. LDH Release Assay

Lactate dehydrogenase (LDH) release was determined using a LDH cytotoxicity assay kit according to the manufacturer's instructions. Cells were treated as described above and after H_2_O_2_ incubation the medium from each well was collected to measure the amount of released LDH from each sample at 490 nm using a microplate reader.

#### 2.2.4. MTT Assay

The MTT assay was performed as previously described [12]. Briefly, H9C2 cells were seeded in flat-bottomed 96-well microplates at a density of 3 × 10^3^ cells in 0.1 mL of culture medium. After 24 h, the cells were incubated in culture medium containing various concentrations of ZCF for the indicated times. Then, the drug-containing medium was removed and replaced by medium containing H_2_O_2_ at a concentration of 0.8 mmol/L, and the cells were then incubated for 4 h. 20 *μ*L of MTT (5 mg/mL) was then added to each well. After incubation for 4 h, 0.15 mL of DMSO was added to stop the reactions. The absorbance values of each well were determined spectrophotometrically at 490 nm using a microplate reader.

#### 2.2.5. Determination of Mitochondrial Membrane Potential (∆Ψm)

JC-1, a dual-emission potential-sensitive probe, was used to measure mitochondrial membrane potential. H9C2 cells were harvested after treatment and incubated with JC-1 staining buffer for 20 min at 37°C in the dark. Cells were washed twice with washing buffer, resuspended in 1 mL washing buffer and analyzed using a microplate reader. ∆ Ψm was determined by the ratiometric analysis of red fluorescence emitted by JC1 aggregates* versus* green fluorescence emitted by the JC-1 monomer in the cells.

#### 2.2.6. Detection of Intracellular Reactive Oxygen Species (ROS)

After H_2_O_2_ treatment, cells were washed 3 times with serum-free culture medium and then 0.5 mL of dichloro-dihydro-fluorescein diacetate (DCFH-DA) dye (10 *μ*mol/L, diluted with PBS) was added to each hole. After incubating for 20 min at 37°C, cells were washed three times with PBS, trypsinized, and suspended in 200 *μ*L PBS. The orifice plates containing a cell suspension were placed on the black transparent bottom and subjected to fluorescence microscope radiography using an excitation wavelength of 488 nm and an emission wavelength of 525 nm. The mean fluorescence intensity (MFI) of DCFH-DA was positively correlated to the ROS level in the cells.

#### 2.2.7. *Determination of Intracellular Na*^+^*/K*^+^*-ATPase Activity and ATP Content*

H_2_O_2_ treated cells were washed twice with saline solution and subjected to ice bathing cracking in the presence of 200 *μ*L NP-40 cracking liquid, according to the manufacturer's instructions for the ultra-micro Na^+^/K^+^-ATPase enzyme kit (Jiancheng Biological Engineering Research Institute of Nanjing, Nanjing, China). To measure intracellular Na^+^/K^+^-ATPase activity, the phosphorus fixed method was used. The BCA protein assay kit (Enhanced Type Blue Sky Biotechnology Research Institute, Haimen, Jiangsu Province of China) was used to determine protein concentrations. To detect intracellular ATP levels, a chemiluminescence method was used that employed the Synergy2 multifunctional enzyme marker (Boteng Instrument Co., Ltd. (BIO-TEK).

### 2.3. Effects of ZCF in an* In Situ* Myocardial Ischemia-Reperfusion Injury Rat Model

#### 2.3.1. Animals and Groupings

Forty-eight male and female Sprague-Dawley rats (8 weeks old, average weight 250 ± 50 g) were provided by the Experimental Animal Center of Zhejiang Province Culturing. This study was approved by the ethics committee of the Zhejiang Academy of Medical Sciences and Xinjiang Institute of Materia Medical, and all procedures involving animals were performed in accordance with the guidelines for humane treatment of laboratory animals from the committee for care and use of laboratory animals (No. 2015RCK0105)

#### 2.3.2. Establishment of the Model

Rats were randomly divided into 6 groups: a positive control group; the sham-operated group; model group; and the ZCF groups with low (75 mg/kg), medium (150 mg/kg), and high concentration (300 mg/kg) treatments, respectively. Rats in the positive control group were treated with isosorbide mononitrate (10 mg/kg) to dilate blood vessels. Rats in each ZCF group received ZCF at the indicated concentration by intragastric administration for 7 days. Rats in the sham-operated and the model group received equal volumes of distilled water. Subsequently, rats were anesthetized with 10% chloral hydrate by intraperitoneal injection, positioned on the operating table, and disinfected regularly. Neck tracheotomy was performed and a breathing machine was connected with a tidal volume of 3 mL/100 g at a frequency of 70 breaths/min. A 2 cm skin incision was made along the midline of the left collarbone and the chest was opened between the third and fourth ribs to expose the heart. To cause myocardial ischemia, the pericardium was incised and the left anterior descending coronary artery at the site of the coronary vein between the arterial cone and the left auricle ligatured. Myocardial ischemia was evaluated by an increase or a decrease in the ECG ST segment and/or T wave. After being subjected to ischemia for 30 min, the blood supply of the left anterior descending coronary artery was restored and reperfusion was performed for 60 min to establish the model.

#### 2.3.3. Determination of Related Indices and Calculation of the Myocardial Ischemia Infarction Area by TTC Dyeing

After reperfusion, the full-thickness of the left ventricular myocardial was used to prepare 10% homogenates in an ice bath, followed by cryogenic centrifugation. The supernatant was cryopreserved at -80°C. The MDA and SOD activities were measured according to the manufacturer's protocols (Jiancheng Biological Engineering Research Institute of Nanjing, Nanjing, China).

The myocardial ischemia infarction area was measured using the triphenyl tetrazolium chloride (TTC) dye method. Following reperfusion, the left anterior descending coronary artery was ligatured again and 2 mL of 2% Evans Blue (EB) solution was injected through the jugular vein. As soon as the oral and peripheral limb skin was stained blue, the heart was quickly excised and rinsed with cold saline to remove residual dye, followed by cryopreservation at -80°C. The frozen heart was cut into 5 slices of equal thicknesses, vertically, from the cardiac apex to the cardiac base along the atrioventricular groove. Tissue sections were incubated in 1% TTC solution for 15 min at 37°C and fixed in 4% formaldehyde overnight. Normal muscle was stained blue, ischemia myocardial muscle without infarction was stained red, and ischemic myocardial muscle with infarction was stained white. Tissue sections were subjected to digital camera imaging and analyzed using image analysis software (Motic Adverse 3.2 imaging analysis system, Motic (Xiamen) Electric Group Co., Ltd.) to measure the size of the blue, red and white areas. The size of the entire left ventricle (LV) area was defined by the sum of the blue, white and red area sizes. The ischemia area (the area at risk, AAR) was defined as the sum of the red and white areas, while the infarction area (IS) was defined as the white area size. The sizes of the ischemia and infarction areas were expressed as follows:

(1) %  of  ischemia  sizeAARLV=ischemiaLV  size×100%%  of  infarction  sizeISAAR=infarction  sizeischemia  size×100%

#### 2.3.4. Histopathological and Morphological Observations of Myocardial Tissue in the Myocardial Ischemia-Reperfusion Injury Rat Model

After the ischemia-reperfusion procedure, the myocardial tissue was excised from the cardiac apex, fixed in formaldehyde and stained with hematoxylin and eosin (H&E). The tissue samples were thinly sliced for histopathological and morphological observation and viewed with a light microscope (OLYMPUS BX-41).

The criteria used for pathological rating of tissue damage were: (0) neatly arranged muscle fibers, transparent cross striations, clear nuclei, and no cell swelling; (I) discrete myocardial necrosis mainly with coagulation necrosis confined to the endocardial area; (II) patchy distribution of myocardial necrosis foci, no connection between lesions which involved the full-thickness of the heart muscle wall; (III) extensive myocardial necrosis, mutual connection between lesions which involved the full-thickness of the heart wall; (IV) widespread damage throughout the heart including extensive necrosis-like infarction with occasional acute aneurysm or mural thrombus.

### 2.4. Statistical Analysis

SPSS ver. 13.0 software was used for statistical analyses. Data are expressed as the mean ± SEM. All experiments with cultured cells, including preparation and measurements, were repeated at least 3 times. For animal experiments, measurements were repeated at least 8 times and average values of data calculated. Statistical analyses were performed with an unpaired Student's* t*-test after the demonstration of homogeneity of variance with an F test or a one-way ANOVA for more than two groups.* P* values < 0.05 were considered to be statistically significant.

## 3. Results

### 3.1. Effect of ZCF on H9C2 Cell Viability

**Figure 1:**
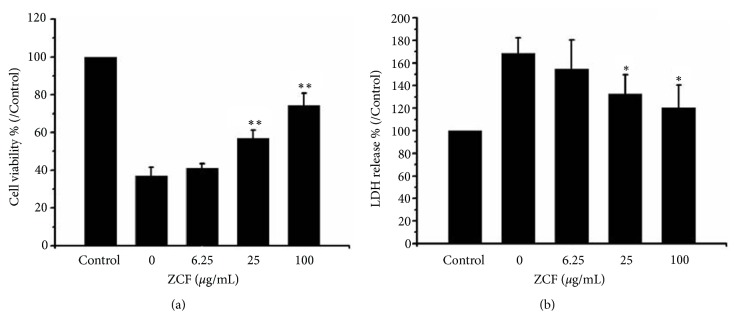
Effects of ZCF preconditioning on cell viability against H_2_O_2_-induced injury. (a) Cell viability assay. (b) Lactate dehydrogenase release was measured in the cell supernatant. Data are representative of 3 independent experiments and presented as means ± SD. *∗P *< 0.05, *∗∗P *< 0.01 compared to the H_2_O_2_ group.

Cell viability determined by MTT assay was higher in the preconditioned ZCF (6.25, 25, and 100 *μ*g/mL) groups* versus* the H_2_O_2_-treated group (I/R cells). As shown in Figure 1(a), ZCF effectively protected H9C2 cell viability in a concentration-dependent manner. In cells treated with ZCF at medium (25 *μ*g/mL) and high (100 *μ*g/mL) concentrations, the cell viability was 57.0 ± 4.2% and 74.4 ± 6.4%, respectively, which was significantly higher than in I/R model cells (37.1 ± 4.5%,* P* < 0.01).

### 3.2. Effects of ZCF on LDH Release in H_2_O_2_ Injured H9C2 Cells

As a necrosis marker, we measured the levels of LDH released into the culture media, which represents disruption of the plasma membrane. As shown in Figure 1(b), LDH leakage of I/R model cells significantly increased compared to the control group (168.7 ± 13.8%* versus* 100%). Reduced LDH release was observed in cells treated with ZCF at medium (25 *μ*g/mL) and high (100 *μ*g/mL) concentrations (132.8 ± 16.8% and 120.6 ± 19.9%), respectively, which was decreased significantly compared with those of I/R model cells (168.7 ± 13.8%,* P* < 0.05).

### 3.3. Effects of ZCF on the Change of ∆Ψm in H_2_O_2_ Injured H9C2 Cells

**Figure 2:**
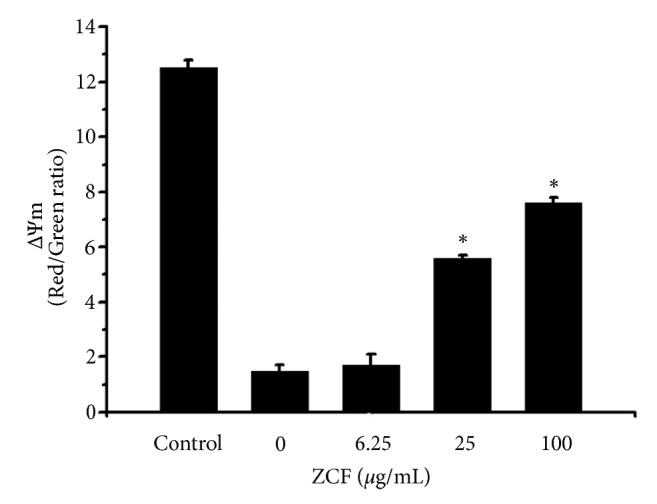
∆Ψm in H9C2 cells cultured under ZCF preconditioning after 24 h was determined by using JC-1 as indicated in the* Methods*. Ratiometric analysis of the fluorescence of JC1-aggregated form (red)* versus* JC1-free form (green) from 3 different experiments performed in duplicate. *∗P *< 0.05 compared to the H_2_O_2_ group.

As mitochondrial membrane potential (∆ Ψm) is the central bioenergetic parameter controlling the generation of ROS, we examined the ∆ Ψm signal by using 5,5,6,6-tetrachloro-1,1,3,3-tetraethylbenzimidalolylcarbocyanine iodide (JC-1) in H9C2 cells treated with either ZCF and H_2_O_2_ or H_2_O_2_ alone. The effects of ZCF on changes of ∆ Ψm in H9C2 cells are shown in Figure 2. H9C2 cells exposure to H_2_O_2_ led to a remarkable drop of 1.5 ± 0.2 in ∆ Ψm compared to the control group (12.5 ± 0.3). However, when H9C2 cells were pretreated with ZCF, the mean values of ∆ Ψm increased to 1.7 ± 0.4, 5.6 ± 0.1 and 7.6 ± 0.2, respectively, and that the effect of ZCF treatment was concentration-dependent.

### 3.4. ZCF Attenuated Overproduction of ROS Caused by H_2_O_2_ Treatment in H9C2 Cardiomyocytes

**Figure 3:**
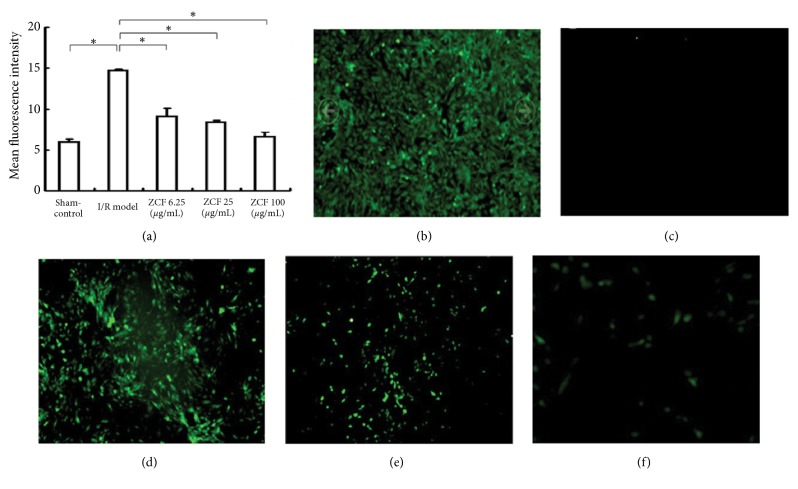
ZCF attenuates overproduction of ROS induced by H_2_O_2_ in H9C2 cells. (a) Fluorescence intensities in control cells, I/R cells and cells pretreated with ZCF at the indicated concentrations. *∗P* < 0.05; B-F: Fluorescence microscope radiography of I/R cells (b), control cells (c) and cells treated with ZCF at concentrations of 6.25 *μ*g/mL (d), 25 *μ*g/mL (e) and 100 *μ*g/mL (f).

H9C2 myocardial cells were treated with ZCF at the indicated concentrations and then exposed to 0.8 mmol/L H_2_O_2_ for 4 h to produce ROS, which is one measure of ischemia-reperfusion injury. The intracellular levels of ROS were measured with a microscopic detection system for fluorescence intensity. No significant fluorescence signals were observed in untreated control cells. However, the fluorescence intensity increased in cells treated with H_2_O_2_ (I/R cells), demonstrating an increase in ROS levels. The difference in the average fluorescence intensity between control and I/R cells was significant (*P* < 0.001). When ZCF pretreated cells were exposed to H_2_O_2_, the fluorescence intensity decreased in a ZCF concentration-dependent manner (Figures 3(a)–3(f)), indicating that the levels of intracellular ROS were significantly lower compared to control I/R cells and that the effect of ZCF treatment was concentration-dependent.

### 3.5. Effects of ZCF on Intracellular Na^+^/K^+^-ATPase Activity and the ATP Content of H9C2 Cells

To evaluate the ZCF protective effect on energy metabolism, the activity of Na^+^/K^+^-ATPase and the content of ATP were measured in H9C2 I/R model cells using an ultra-micro assay. The Na^+^/K^+^-ATPase activity in I/R cells was lower than in the control group (22.06 ± 6.06* versus *37.93 ± 3.50,* P *< 0.001). However, in ZCF pretreated cells, the Na^+^/K^+^-ATPase activity was significantly increased in a concentration-dependent manner. Cells treated with a high concentration (100 *μ*g/mL) of ZCF (ZCFH group) showed Na^+^/K^+^-ATPase activity (33.89 ± 5.00 U/mg^−1^ protein) almost as high as that of cells in the control group (*P* = 0.18), which was significantly higher than in the I/R group (*P *= 0.010, Table 1).

### 3.6. Effects of ZCF on Intracellular Levels of ATP in H9C2 Cells

H_2_O_2_ treatment of H9C2 cells decreased the intracellular ATP concentration to 2.77 ± 1.13 nmol/mg, which was significantly difference from the control group (*P* < 0.001). When cells were pretreated with increasing concentrations of ZCF, a decrease in the ATP content was found, which was proportional to the applied ZCF concentration. In cells treated with ZCF at medium (25 *μ*g/mL) and high (100 *μ*g/mL) concentrations (ZCFM and ZCFH groups), the ATP content was 11.74 ± 1.21 and 23.09 ± 3.4 nmol/mg, respectively, which was significantly higher than in I/R model cells (*P* < 0.001, Table 2).

### 3.7. Effects of ZCF on Myocardial Ischemia and the Infarction Area in the Ischemia-Reperfusion* In Situ *Rat Model

**Figure 4:**
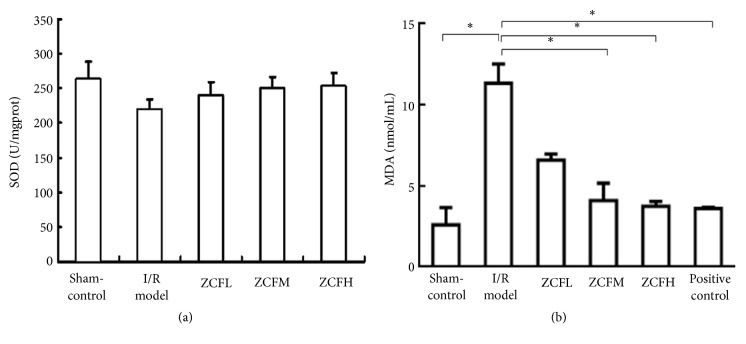
Effects of ZCF on SOD (a) and MDA (b) activity in myocardial tissue of rat hearts after ischemia-reperfusion* in situ* (*n* = 8). *∗P* < 0.05.

After being subjected to the ischemia-reperfusion procedure* in situ*, rat hearts were homogenized and the SOD content was measured. The results showed that SOD activity was significantly decreased in the I/R control group compared to the normal control group (*P* < 0.5). In all ZCF groups, SOD activity was higher than in the I/R group, an effect that was concentration-dependent. Differences found between the ZCFH and the I/R group and the ZCFM group and the I/R group were statistically significant (*P* < 0.01, Figure 4(a)).

In contrast, the MDA content was markedly increased in myocardial tissue after ischemia-reperfusion (I/R model group). The increase was significant compared to the sham operation group (*P* < 0.001). However, the increase in the MDA content in the ZCFH and the ZCFM groups was clearly inhibited and the differences between these groups and the I/R model group were statistically significant (*P* < 0.001, Figure 4(b)).

### 3.8. Optical Microscopic Inspection of the Ischemic Heart

**Figure 5:**
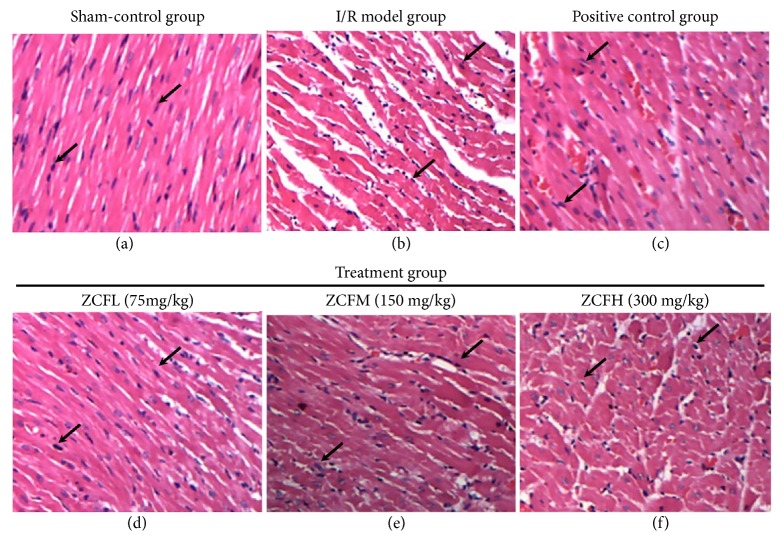
Pathological changes of myocardium tissue after ischemia-reperfusion and the effects of ZCF in an* in situ* rat model (HE × 100). (a) Sham-control group; (b) I/R model group. (c) Positive control group. (d) ZCFL (75 mg/kg). (e) ZCFM (150 mg/kg). (f) ZCFH (300 mg/kg).

Myocardial tissue samples were prepared and stained with H&E followed by examination using an optical microscope. Tissue from the sham-operated group showed neatly arranged myocardial fibers, an oval-shaped nucleus, clear textured bundles, no cell swelling, and no area of necrosis. Tissue from the I/R group exhibited irregularly arranged and undulated bent myocardial fibers with widened gaps, nucleus pyknosis, myocardial cell lesion, and necrosis. In contrast, neatly arranged myocardial fibers, clear textured bundles, no cell swelling, and no necrosis were observed in tissue from all ZCF groups and the active control group, treated with isosorbide mononitrate (Figure 5). Measurements using a digital imaging system demonstrated that the percentage of the myocardial ischemia area was significantly higher in tissue sampled from the I/R model group compared to the sham group. However, tissue from the ZCF-treated groups showed less ischemia area compared to the I/R model group (*P* < 0.001, Table 3).

## 4. Discussion

The leaves, flowers, and stems of the ZC plant are frequently used in folk medicine for the treatment of hypertension, coronary heart disease, and other cardiovascular and cerebrovascular diseases. To determine whether ZCF-treated H9C2 cells were resistant to H_2_O_2_-induced injury, the viability of treated cells was evaluated using MTT and LDH release assays. Cell viability was assessed by use of the MTT assay in the presence of graded concentrations of ZCF (6.25, 25, and 100 *μ*g/mL). The results indicated that 25 *μ*g/mL and 100 *μ*g/mL ZCF significantly minimized H_2_O_2_-induced cell death compared to I/R model cells. The leakage of LDH into culture medium from treatment groups was monitored. LDH has been regarded as an indicator of the degree of cell death. The level of LDH was significantly increased after H_2_O_2_ treatment, whereas when ZCF was added to the culture medium before H_2_O_2_ treatment, LDH leakage from the treatment groups was suppressed. These findings indicate that, in our experimental setting, ZCF is effective in inhibiting H_2_O_2_-induced injury.

H_2_O_2_ disrupts redox homeostasis and accumulates excessive ROS. As the major site of ROS production, the function of mitochondria, including mitochondrial membrane potential, is then severely affected. Our* in vitro *results confirmed a ZCF-dependent increase of ∆ Ψm in I/R model cell mitochondria. We have demonstrated that H_2_O_2_ treatment leads to the generation of substantial quantities of ROS in H9C2 rat myocardial cells and that ZCF antagonized ROS generation in a concentration-dependent manner (Figures 3(a)–3(f)). We found that ZCF pretreatment of H9C2 myocardial cells significantly prevented the reduction of the intracellular activity of Na^+^/K^+^-ATPase and ATP content (Tables 1 and 2). The production of intracellular ROS is a well-known characteristic of ischemia-reperfusion injury. The activity of Na^+^/K^+^-ATPase and the content of ATP are the major indices of energy metabolism, which is a crucial process in myocardial cells. In the event of myocardial ischemia, myocardial energy metabolism is most significantly affected [13]. The shortage of energy supply is one of the pathophysiological processes involved in cardiac failure and shock caused by myocardial ischemia [14]. Our results have demonstrated that ZCF positively affected cardiac energy metabolism during ROS exposure.

The ATP enzyme is one of the proteases located in the cell membrane and plays a vital role in material transport, energy conversion, and information transfer [15]. In addition, Na^+^/K^+^-ATPase has a dual function: as an enzyme it catalyzes the hydrolysis of ATP and as a carrier it releases energy. The energy release reverses the chemical gradient for ion transport, by which the normal distribution of Na^+^ and K^+^ is maintained in the cell. Thus, Na^+^/K^+^-ATPase is essential for the maintenance of normal cell functions and internal environment stability [16].

A variety of myocardial reperfusion treatments for acute myocardial infarction cause ischemia-reperfusion injury (I/R), which results in a common pathophysiological change and often compromises the recovery of heart function or sometimes causes serious complications such as arrhythmia, myocardial energy metabolism dysfunction, and cardiac muscle inhibition [17–19].

In the hearts of our* in situ *I/R rat experiment, we found that SOD activity was decreased; in contrast MDA content was increased during cardiac ischemia-reperfusion in the I/R groups, thus revealing a substantial production of free radicals. Compared with the I/R model control group, hearts from the ZCFH group showed significantly higher levels of SOD activity and lower MDA content after ischemia-reperfusion, indicating that ZCF antagonized the action of free radicals and helped to prevent lipid peroxidation, which was reflected in essentially reduced morphological heart muscle cell damage (Figure 4).

## 5. Conclusions

ZCF protected myocardial cells from ischemia-reperfusion injury by removing oxygen free radicals, which resulted in reduced lipid peroxidation, restoration of the cardiac energy level and function, and maintenance of the integrity of the heart cell membranes.

## Figures and Tables

**Table 1:** Effects of ZCF on intracellular Na^+^/K^+^-ATPase activity in H9C2 cells.

Group	ZCF (*μ*g/mL)	Na^+^/K^+^- ATPase activity (U/mg)
Control	—	37.93 ± 3.50
I/R	—	22.06 ± 6.06^###^
ZCFL	6.25	29.22 ± 6.92
ZCFM	25	31.00 ± 7.93
ZCFH	100	33.89 ± 5.00^*∗∗*^

^###^Statistical comparison with the control (*P* < 0.001).

^*∗∗*^Statistical comparison with the control (*P* < 0.01).

**Table 2:** Effects of ZCF on the intracellular ATP content in H9C2 cells.

Group	ZCF (*μ*g/mL)	ATP content (nmol/mg protein)
Control	—	30.29 ± 4.38
I/R	—	2.77 ± 1.13^###^
ZCFL	6.25	4.19 ± 0.63
ZCFM	25	11.74 ± 1.21^*∗∗∗*^
ZCFH	100	23.09 ± 3.49^*∗∗∗*^

^###^ Statistical comparison with the control (*P* < 0.001).

^*∗∗∗*^ Statistical comparison with the I/R group (*P* < 0.001).

**Table 3:** Effects of ZCF on the myocardial ischemia and infarction area in the *in situ* ischemia-reperfusion rat model (*n* = 8).

Group	ZCF (mg/kg)	Myocardial ischemic area (%)	Myocardial infarct area (%)
Sham	—	16.2 ± 5.0	3.0 ± 0.3
I/R	—	58.0 ± 6.1^1)^	10.8 ± 1.9 ^1)^
ZCFH	300	43.3 ± 4.5^2)^	8.5 ± 1.2 ^2)^
ZCFM	150	47.2 ± 4.3^2)^	9.6 ± 0.5
ZCFL	75	53.6 ± 6.2^2)^	10.2 ± 0.8
Active control	10.0	45.4 ± 3.3^2)^	6.4 ± 0.5 ^2)^

Note: comparison with the Sham group, ^1)^*P* < 0.001; comparison with the I/R group, ^2)^*P* < 0.05.

## Data Availability

The data used to support the findings of this study are available from the corresponding author upon request.

## Abbreviations

ZCF: * Ziziphora clinopodioides Lam*. Flavonoids
I/R: Ischemia-reperfusion
LDH: Lactate dehydrogenase
ROS: Reactive oxygen species
ZCFL: ZCF high concentration
ZCFM: ZCF medium concentration
ZCFH: ZCF high concentration
SOD: Superoxide dismutase
MDA: Malondialdehyde
ZC: * Ziziphora clinopodioides*
ROCCs: Receptor-operated Ca^2+^ channels
DCFH-DA: Dichloro-dihydro-fluorescein diacetate
MFI: Mean fluorescence intensity
TTC: Triphenyl tetrazolium chloride
EB: Evans Blue
LV: Left ventricle
AAR: Area at risk
IS: Infarction area
H&E: Hematoxylin and eosin
JC-1: 5,5,6,6-tetrachloro-1,1,3,3-tetraethyl-benzimidazolyl-carbocyanine iodide.
